# Dialectical behaviour therapy for treating adults and adolescents with emotional and behavioural dysregulation: study protocol of a coordinated implementation in a publicly funded health service

**DOI:** 10.1186/s12888-018-1627-9

**Published:** 2018-02-26

**Authors:** Daniel Flynn, Mary Kells, Mary Joyce, Catalina Suarez, Conall Gillespie

**Affiliations:** 1 Cork Mental Health Service, Cork Kerry Community Healthcare, Health Service Executive, St Finbarr’s Hospital, Cork, Ireland; 2 National Suicide Research Foundation, Western Gateway Building, University College Cork, Cork, Ireland; 3 Cork Mental Health Service, Cork Kerry Community Healthcare, Health Service Executive, Inniscarraig House, Western Road, Cork, Ireland

**Keywords:** Dialectical behaviour therapy, Borderline personality disorder, Effectiveness, Implementation, Economic cost, Adults, Adolescents, Public health service, Community settings

## Abstract

**Background:**

In the Republic of Ireland, borderline personality disorder (BPD) is a feature of approximately 11–20% of clinical presentations to outpatient clinics within mental health services. These estimates are similar to other countries including the UK and USA. Dialectical behaviour therapy (DBT) is an intervention with a growing body of evidence that demonstrates its efficacy in treating individuals diagnosed with BPD. While a number of randomised controlled trials (RCTs) have demonstrated the efficacy of DBT, there is limited research which evaluates the effectiveness of this model when applied to real world settings. Funding was secured to co-ordinate DBT training in public community-based mental health services across Ireland. As no other study has evaluated a co-ordinated national implementation of DBT, the current study proposes to investigate the effectiveness of DBT in both adult and child/adolescent community mental health services across Ireland, evaluate the coordinated implementation of DBT at a national level, and complete a comprehensive economic evaluation comparing DBT versus treatment-as-usual.

**Methods/ design:**

This study takes the form of a quasi-experimental design. Individuals attending community mental health services who meet criteria for participation in the DBT programme will be allocated to the intervention group. Individuals who live in areas in Ireland where DBT is not yet available, and individuals who choose not to participate in the intervention, will be invited to participate in a treatment-as-usual comparison group. Self-report clinical measures and health service use questionnaires for DBT participants (and parent/guardians as appropriate) will be administered at pre-, mid- and post-intervention, as well as follow-up for participants who complete the intervention. Survey and interview data for DBT therapists will be gathered at three time points: prior to DBT training, 6 months after teams begin delivery of the intervention, and 2 years following training completion.

**Discussion:**

It is anticipated that the results of this study will provide evidence for the effectiveness of DBT for patients, and report on recommendations regarding best practice guidelines for implementation of DBT and its economic merit in a publicly funded service.

**Trial registration:**

ClinicalTrials.gov ID: NCT03180541; Registered June 7th 2017 ‘retrospectively registered’.

## Background

Borderline personality disorder (BPD) is a mental health diagnosis characterised by a pervasive pattern of instability of interpersonal relationships, self-image, affect, and marked impulsivity [1]. BPD typically features patterns of cognitive, emotional and behavioural dysregulation that often manifests in self-harm and suicidal behaviours [2]. BPD is recognised as one of the most distressing disorders for individuals and their families, and the most difficult for clinicians to treat. The prevalence of BPD in the general population is reported to be between 0.7% and 1% [3–5]. 10% of outpatients and up to 20% of psychiatric inpatients have this disorder [1]. In the Republic of Ireland, it is estimated that BPD is a feature of 11–20% of clinical presentations to outpatient clinics within mental health services [6]. This is similar to what has been recorded in other countries including the United Kingdom [7], North America [8] and other parts of Europe (e.g. Denmark [9]).

Given the prevalence rates of BPD, there is a growing interest in providing evidence-based treatment for individuals with emotional and behavioural dysregulation. Multiple treatments such as dialectical behaviour therapy [10–12], schema therapy [13], mentalisation based therapy [14], and transference focused psychotherapy [15] have been developed for treating BPD. Dialectical behaviour therapy (DBT) is the most researched treatment option with more than a dozen randomised controlled trials (e.g. [16–18]) which have investigated its efficacy at multiple independent sites [19, 20]. Participation in DBT is associated with reductions in a range of difficulties reported by individuals with BPD including: suicidal behaviour [17, 21–23], suicidal ideation [24, 25], BPD symptoms [26], hopelessness [24] and depression [25, 26]. It has also been associated with improved adjustment [22] and quality of life [23, 26], as well as reduced health service utilisation and/or inpatient psychiatric days [21, 23, 25, 26]. A recent systematic review of randomised studies has shown that DBT is significantly better than treatment-as-usual in terms of leading to reductions in self-harm, decreases in ineffective expression of anger and improvement in general functioning [27]. Although the number of randomised controlled trials for the other listed treatments is still limited, there is an emerging body of evidence highlighting their benefits.

While DBT was initially developed for adults with a diagnosis of BPD, in more recent years, this model has been adapted to make it more developmentally appropriate for adolescents presenting with borderline personality traits such as emotional dysregulation and self-harm [28]. DBT for adolescents (DBT-A) utilizes a similar format to standard DBT where it includes individual therapy sessions, group skills training sessions, phone coaching and weekly consultation meetings for the DBT therapists. However, DBT-A also considers systemic intervention where parent/guardians attend group skills training with the adolescent. The DBT-A programme is also shorter in length and is typically delivered as a 16 week programme. While the research evidence for DBT-A is still in its infancy, reported outcomes are encouraging. To date, one randomised controlled trial found DBT-A to be superior to enhanced usual care in reducing self-harm, suicidal ideation and depressive symptoms [29].

While the outlined research studies have demonstrated the efficacy of DBT in treating BPD in controlled settings, there is a dearth of published research reporting on the effectiveness of DBT in publicly funded community mental health settings. The few studies that have been conducted in community settings have focused on a 6 month DBT programme for adults (e.g. [30, 31]) or have reported on cluster B personality presentations, including but not focusing exclusively on BPD [32]. A number of studies have also highlighted limitations with regard to small sample sizes (e.g. [33, 34]). Comtois, Elwood, Holdraft, Smith and Simpson [35] report on the effectiveness of DBT in a community mental health centre; however, the weekly skills groups were delivered in two 90-min sessions, and the study trial also offered individual DBT case management, both of which are different to the standard DBT programme.

In the Republic of Ireland, an Expert Group on Mental Health published a government policy framework for publicly funded community mental health services which recommended DBT as an evidence-based treatment for people with BPD [6]. As well as being endorsed by the Irish expert group on mental health, DBT has been recommended by the American Psychiatric Association [36] and more recently by the NHS National Institute for Health and Clinical Excellence (NICE [37]) as being a part of any comprehensive treatment programme for patients with BPD and co-morbid presentations.

In line with best practice guidelines, and national mental health policy frameworks, a number of community services in Ireland endeavoured to establish DBT programmes in their locality. Prior to 2013, any such teams were driven by clinician interest and in many instances, failed to sustain as a result of systemic issues. Such issues included lack of management support and funding for training given that DBT had not been listed as a mandated treatment in Irish national health service plans. International research (e.g. [38, 39]) also highlight a number of factors that can impact on effective implementation of DBT. Examples of such barriers include lack of support from public mental health authorities and programme leaders, and absence of organisational support (including staff turnover, and funding for administration, training and supervision). Given positive research outcomes on effectiveness of DBT in a separate study across four sites in the south of Ireland [40], a National Office for Suicide Prevention (established specifically to drive suicide and self-harm prevention strategies in the public health service in Ireland) agreed to fund and support a project team to coordinate a multi-site implementation of DBT in community settings at a national level [41]. As a result, the National DBT Project, Ireland was established in 2013.

As no previous research has been conducted on a coordinated national implementation of DBT in a publically funded mental health system, it was deemed appropriate and necessary to comprehensively evaluate this implementation effort. The research study has three primary aims; first, to assess if individuals who participate in the DBT intervention will achieve positive outcomes when the intervention is delivered as part of a coordinated, multi-site implementation; second, to investigate if a coordinated implementation which addresses known implementation barriers will enable sustainable service provision; and third, to investigate whether it is cost effective to implement DBT versus treatment-as-usual in a publicly funded community mental health setting.

## Methods/ design

### Study setting

Ireland’s public health service, the Health Service Executive (HSE) has the responsibility of delivering all public health services in Ireland [42]. Approximately 10% of individuals who experience mental health difficulties require intensive, co-ordinated care which is accessed through public mental health services. This secondary level care encompasses more specialist interventions delivered by mental health practitioners such as psychiatrists, psychologists, mental health nurses and other professionals. The majority of mental health services in Ireland are provided in the community, typically in outpatient settings, day hospitals, day centres and at home [6].

In Ireland, DBT is typically delivered in community based mental health settings in the public health service [41]. Within this context, core multi-disciplinary staff from multiple community mental health teams are seconded from their existing role to train in DBT and offer this intervention as an evidence-based treatment for individuals with BPD attending their local mental health service. Thus, the setting for this study is Community Mental Health Services where participants attend an outpatient community clinic to obtain the intervention (see Flynn, Kells & Joyce [41] for further information on health service structure in Ireland). There are 16 independent sites for this study which cover both urban and rural areas in adult and child/adolescent mental health services.

### Study design

This study takes the form of a multi-site quasi-experimental design with non-equivalent groups. As the setting for this study is the publicly funded health system, staff have a responsibility to treat every individual who presents to the service. As a result, patients attending Community Mental Health Services who meet eligibility criteria for participation in DBT will be offered the intervention. At the onset of this study, there was no alternative system wide evidence-based intervention available for this client group that could have been used for comparison purposes. Additionally, in abiding with ethical guidelines and appropriate care of patients, neither is it possible to withhold treatment for individuals who meet criteria for participation in the intervention. Therefore, it was not possible to randomly allocate participants to a DBT intervention, comparable intervention or control group for the purposes of this study. Given this limitation, a treatment-as-usual comparison group was utilised which comprises of individuals who cannot access DBT in their area or who opt out of DBT as a treatment option (but continue to engage in routine care).

### Participants

#### Patients

##### Inclusion criteria

The inclusion criteria for adults for participation in the study are:

- Diagnosis (or meet criteria for diagnosis) of borderline personality disorder (DSM-IV-TR, [1]) or emotionally unstable personality disorder (ICD-10, [43])
- A persistent pattern of self-harm behaviour or suicidal behaviour, with the most recent episode having occurred within the six months prior to being referred to the intervention
- Will participate in all modes of treatment and have committed to participate in the standard 12-month DBT intervention

The inclusion criteria for adolescents are:

- Demonstrate emotional behavioural disturbance/ emotional dysregulation
- A persistent pattern of self-harm with self-harm behaviour or a suicidal act having occurred within the last 16 weeks or chronic suicidal ideation reported
- The young person and their parent/guardian will participate in the recommended modes of treatment and have committed to participate in the 16-week DBT-A intervention

Patients who have co-morbid axis I and axis II disorders are permitted to participate in this study as are those who are also using medication.

##### Exclusion criteria

The following exclusion criteria for adults and adolescents apply:

- An active psychosis
- Severe developmental delays, cognitive impairment or learning difficulties (that exceed the mild range)
- Substance/ drug dependence, eating disorder or any other mental health issues/behaviour at such a level that it would impede their engaging with any of the modalities of DBT.

#### Treatment-as-usual

Study sites for the treatment-as-usual comparison group will be based in areas where Community Mental Health Teams have expressed an interest in DBT, but are unable to complete training as a result of implementation barriers during the four-year study period. Therefore, patients who reside in areas where the treatment is not yet available, and who are engaged with their local Community Mental Health Service, will be invited to participate in the treatment-as-usual group. Additional treatment-as-usual participants will be patients at intervention sites who have been offered the treatment but have decided not to partake at that time. The same inclusion/exclusion criteria apply for treatment-as-usual participants as for the intervention group except that participants are not in a position to participate in all modes of DBT treatment and/or commit to the full programme.

#### Parent/guardian of adolescent

For DBT programmes which are delivered in child/adolescent mental health services, the adolescent’s parent/guardian accompanies their child for the weekly group skills training session. It should be noted that the parent/guardian does not receive any therapeutic treatment as part of the DBT programme. Miller, Rathus & Linehan [28] recommend the requirement of the same family member attending each week and to make the same attendance commitment as the adolescent. Potential situations where family members may be excluded from the intervention include the following:

- A parent’s work schedule or other obligations make it impossible for him or her to attend
- Estrangement between the parent and the adolescent
- There is such an intense degree of parent-adolescent conflict that their being in a group together would be likely to result in explosive or otherwise therapy-destroying behaviour
- There is an ongoing abusive situation and the adolescent is looking to maintain distance and safety
- A parent has a serious unmanaged mental disorder

Adolescents whose parent/guardian do not attend the weekly group skills training will be retained in the study as long as consent from a parent/guardian has been provided for the adolescent to participate in the research study.

#### DBT therapists

All therapists who receive DBT training as part of the National DBT Project, Ireland will be invited to participate in the study. The structure of DBT teams in Ireland has followed the recommendations of the UK licensed training provider of Intensive Training™ which specify that teams who want to train in DBT must have a minimum of four team members and a maximum of ten.^1^ Each team must have either a clinical/forensic/counselling psychologist OR a person with demonstrable graduate training in behaviour therapy. All teams must be genuine teams i.e. who either are already or have explicit plans to meet together to deliver a comprehensive DBT programme to a group of clients in a single setting e.g. out-patient adult clients. Each individual team member: must be employed by a healthcare organisation that expects them to be seeing clients; must be registered to practice with a regulatory professional body; must commit at least 15 h per week to learning and delivering DBT [44]. At each study centre, members of the DBT team who receive DBT training as part of this project will provide individual therapy to the patient, deliver the weekly group skills sessions, provide phone coaching for clients, and attend weekly team consultation meetings.

### Intervention

Standard DBT for adults is delivered by a team of multidisciplinary mental health professionals, and comprises individual therapy sessions for each patient, group skills training sessions, phone coaching and consultation meetings for the clinicians on the DBT team [11, 12]. In stage 1 of DBT, which focuses on behavioural stabilisation, all treatment modalities are delivered on a weekly basis over the course of a 12 month programme. A summary of the modes and their functions [11] are outlined in Table 1.

**Table 1:** Modes and functions of standard DBT for adults

Mode	Function	Frequency	Duration
Individual Therapy	Motivation for treatment, treatment goals and skills strengthening	Weekly	60 min
Skills Training	Enhance patient capabilities – skills acquisition and strengthening	Weekly	2.5 h
Phone consultation	Assist patients to generalise skills to daily life and in crises	As needed	10–15 min
Team Consultation	Enhance therapists capabilities and motivation to adhere to DBT, peer support and prevent burn-out	Weekly	1.5–2 h

Group skills training is delivered in blocks of three modules which teach mindfulness, distress tolerance, emotion regulation and interpersonal effectiveness. The three modules are delivered over a 24-week period and are then repeated.

DBT-A utilises a similar format to standard DBT and is delivered by a team of multidisciplinary mental health professionals. However, as part of the DBT-A adaptation, the treatment length is reduced from 12 months to 16 weeks [28]. The skills and modules are shortened and the materials are made more developmentally appropriate for adolescents. In DBT-A, each module is only taught once. DBT-A also includes an additional module, Walking the Middle Path, which addresses adolescent and family dilemmas. In addition, parent/guardians are included in the weekly skills groups as part of a multifamily group component.

### Treatment adherence

DBT is a principle rather than protocol driven treatment. It outlines a series of principles to help the practitioner decide on what to do in a given set of circumstances. The principles guide the therapist to being treatment adherent while remaining responsive to individual patient needs [45]. A diary card (which tracks a person’s urges, mood and DBT skill use) is used to help structure the individual therapy session and target which behaviours need a chain and solution analysis. Treatment adherence will be monitored by means of supervision and review of audio recorded individual therapy sessions by supervisors. Feedback on treatment adherence is then provided to the clinician by the supervisor.

### Concomitant care

Typically patients continue with medical and psychiatric treatment but are not engaged in other psychotherapy at the time of the DBT intervention.

### Outcome measures

#### Effectiveness evaluation

Primary outcome measures for patients in this study will directly map onto DBT treatment targets which are:

- reduction of life threatening behaviours
- reduction of treatment interfering behaviours
- reduction of quality of life interfering behaviours
- increase in skill utilisation

More specifically, the treatment target, corresponding measurement variables, and participant groups are outlined in Table 2:

**Table 2:** Treatment targets, measurement method and reporting method for primary outcomes

			Completed by		
Treatment target		Measure	Adults	Adolescents	DBT Therapists
Life threatening behaviours	Self-harm	Self-harm Inventory [58]	✓		
		Client record form^2^			✓
	E.D. visits	Client record form			✓
	Hospital admissions	Client record form			✓
	Suicidal Ideation	Questionnaire for suicidal ideation	✓	✓	
Treatment interfering behaviours	Attendance	Individual therapy/group skills logs^3^			✓
	Use of phone coaching	Phone coaching logs^3^			✓
Quality of life interfering behaviours	Depression	Beck Depression Inventory – II [59] Beck Depression Inventory-Youth [60]	✓	✓	
	Borderline symptoms	Borderline Symptoms Checklist [55]	✓	✓	
	Hopelessness	Beck Hopelessness Scale [61]	✓	✓	
	Quality of life	EQ-5D-5 L [62]	✓	✓	
	Dysfunctional coping	DBT Ways of Coping Checklist [63]	✓	✓	
	Anger	STAXI - 2 [64] STAXI - C/A [65]	✓	✓	
Skill utilisation	Skills use	DBT Ways of Coping Checklist	✓	✓	

^2^Developed by research team in consultation with DBT therapists to systematically gather data pertinent to the Irish public health service. Self-harm behaviour frequency and type, number of Emergency Department visits, and number and duration of acute psychiatric inpatient admissions per patient

^3^Developed by research team and outlined in more detail under Implementation Evaluation

Secondary outcome measures will also be completed by DBT therapists to provide an objective perspective on patient functioning at each time point. The measures completed by DBT therapists for adult participants are the Global Assessment of Functioning [46] and the Health of the Nation Outcome Scales [47]. The corresponding adolescent versions of these scales were used for the adolescent participant group [48, 49].

Constructs relevant for family members of individuals with behavioural and emotional dysregulation are assessed through scales which measure parental stress [50], burden [51] and grief [52].

#### Implementation evaluation

**Fig. 1:**
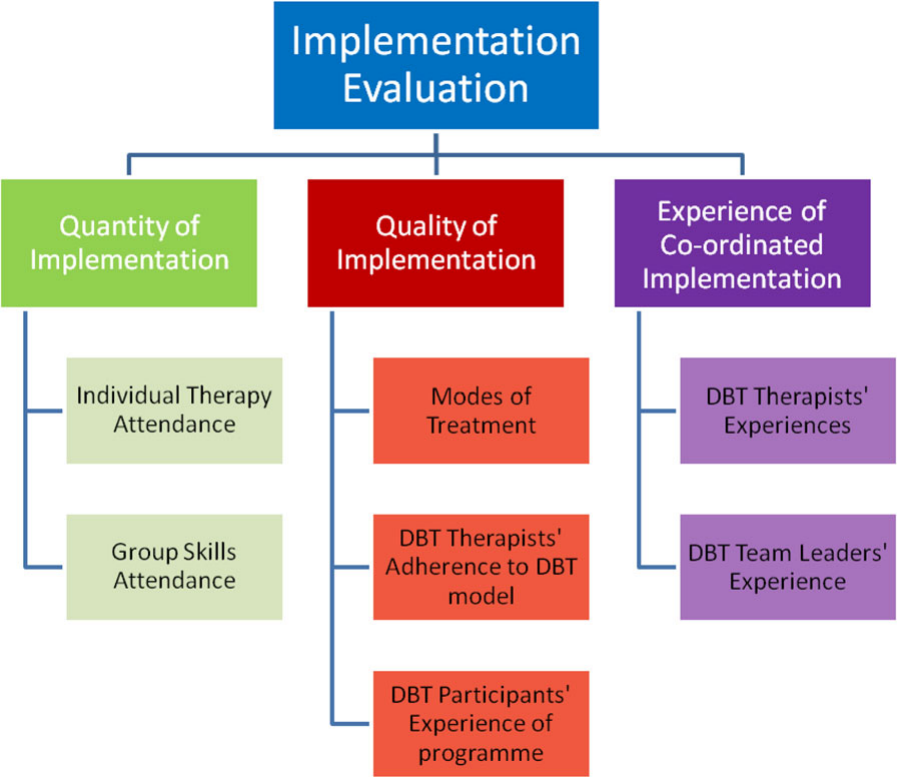
Overview of Implementation Evaluation

The coordinated implementation (Fig. 1) will be evaluated in the following manner:

The quantity of implementation will be measured through individual therapy and group skills attendance logs which are recorded by the DBT therapists on a weekly basis. These DBT Programme Logs developed for the purposes of this study (reporting individual therapy and group skills attendance, self-harm behaviour and urges, and skills use in the last week) will be used to record this information.

The quality of implementation will be measured through the Programme Elements of Treatment Questionnaire (PETQ; [53]). DBT therapist’s adherence to the DBT model will be assessed on an ongoing basis by the expert DBT supervisor working with each team. DBT participants’ experience of the programme will be assessed through a survey which has been specifically developed for the purpose of this study. Survey questions request feedback on overall quality of the intervention, usefulness of content, and effectiveness. Experience of the coordinated implementation will be measured through surveys which have been developed based on international DBT implementation research. The surveys will be used to gather information about DBT therapists’ experiences of the implementation. Survey questions cover areas including training, supervision, implementation facilitators and barriers, and experience of coordinated implementation.

#### Economic evaluation

A client record form (see section on Outcome Measures) has been developed for DBT therapists to track detailed information about service utilisation and resource use by DBT patients. Effectiveness outcome measures (e.g. EQ-5D-5 L and BDI-II) will also be used to inform the economic evaluation.

### Time point for each outcome

#### Effectiveness evaluation time points

There will be different time points for each sample in this study as the treatment length for adult and adolescent patients is different.

For **adult** patients, the treatment length is 12 months in duration, so there will be four time points for assessment of outcome: baseline (during the week prior to the patients’ first group skills training session), 6 months after baseline (end of module 3), 12 months after baseline (end of programme), and 18 months after baseline (6 months after programme completion).

For **adolescent** patients, the treatment length is 16 weeks in duration, so there will be three time points for assessment of outcome: baseline (during the week prior to the patients’ first group skills training session), 16 weeks after baseline (end of programme), and 32 weeks after baseline (16 weeks after programme completion).

#### Implementation evaluation time points

The quantity of the implementation is monitored weekly through recording of individual therapy and group skills weekly attendance logs completed by the DBT therapists at each site.

The quality of the implementation is measured through the PETQ which will be administered to team leaders for completion two years after teams complete Intensive Training Part I. DBT therapists’ adherence is assessed on an ongoing basis through expert supervision. To assess participant’s experience of the DBT programme, a survey is administered at time points 2, 3 and 4 for adults (alongside self-report outcome measures) and time points 2 and 3 for adolescents (alongside self-report outcome measures).

The experience of the coordinated implementation will be measured by administering surveys to therapists at three time points: prior to attending Intensive Training Part I, 6 months after teams begin delivery of their first DBT programme, and 2 years following Intensive Training Part I.

#### Economic evaluation time points

For **adult** patients, the treatment length is 12 months in duration, so there will be four time points for assessment of outcome: baseline (pertaining to 6 months before the start of the programme), end of module 3 (pertaining to the first 6 months of the programme), end of programme (pertaining to the second 6 months of the programme), and 6 months after programme completion (pertaining to the 6 months following completion of the programme).

For adolescent patients, the treatment length is 16 weeks in duration, so there will be three time points for assessment of outcome: baseline (pertaining to 16 weeks before the start of the programme), end of programme (pertaining to the 16 weeks of the programme), and 32 weeks after baseline (pertaining to the 16 weeks following completion of the programme).

### Sample size

It is anticipated that there will be a total of 442 participants across 16 sites in this research study over a four year period. Of the 312, it is estimated that 120 will be adults with a primary diagnosis of BPD attending Adult Mental Health Services across eight study sites. It is estimated that 96 participants will be adolescents with emotional and behavioural dysregulation (emerging borderline personality presentations) across eight study sites. As the adolescent’s caregiver also attends the skills training sessions, there will also be up to 96 parent/guardian participants. All DBT therapists (approximately 130 clinicians) who train as part of the National DBT Project, Ireland will be invited to participate in the research study.

The power calculation is based on the aim of identifying if the intervention is effective across multiple sites as part of a coordinated national implementation with adults and adolescents. It was decided to use a medium effect size of 0.5 according to Cohen [54] for the power calculation with an alpha level of 0.05. The primary outcome measure chosen for the power calculation is the Borderline Symptoms Checklist [55]. With an anticipated sample size of eight clusters per adult group with 15 patients per cluster, and an intracluster correlation coefficient of 0.01, the power to detect change over time is 92%. With an anticipated sample size of eight clusters per adolescent group with 12 patients per cluster, and an intracluster correlation coefficient of 0.01, the power to detect change over time is 86% [56].

### Recruitment/procedure

Newly established teams who train as part of the National DBT Project, Ireland will be requested to inform the researchers of the start date of their DBT programme. All individuals who partake in the DBT programme at each of the 16 sites between February 2014 and February 2016 will be invited to participate in the study. A group data collection session will take place at each time-point at each of the study sites where a member of the research team will facilitate data collection. Data collection will be scheduled in advance with the DBT team at each location. It is anticipated that this will take place at the beginning of the first skills training session that is delivered as part of the intervention. Prior to visiting each site, each DBT team will be provided with Participant Information Leaflets to distribute to patients to orientate them towards the research study. When the researcher attends for data collection, patients will have had time to read the Participant Information Leaflet and will have an opportunity to ask any questions regarding the research study. It will be outlined that while participation in the study is confidential, there is a limit to confidentiality. In order to maintain safety of patients, the researchers will conduct a risk assessment following data collection at each site, the results of which are communicated to the DBT therapist(s) present. Participants who are unable to attend the group data collection session but who have agreed to participate in the study will be asked to complete the battery of measures at their next individual therapy session. These participants will sign the consent form, complete the measures and place the completed measures in a sealed envelope to be collected by the research team. In such cases, the protocol outlines that DBT therapists will review with the patient, only their answers to the risk assessment items, prior to securely storing the completed measures.

For follow-up data collection, participants will be invited to attend a group data collection session with a member of the research team, complete measures with their DBT therapist or complete measures at home in which case, measures will be posted directly to them by the research team. If participants choose to complete the measures at home, participants will first be asked to provide consent for their local General Practitioner to be contacted should self-harm or suicide risk be identified upon completion of the measures.

### Data analysis

T tests and analyses of variance will be used to assess potential baseline difference in the outcome measures. Linear mixed-effects models will be used to estimate change utilising data available from participants at all time points. These models may be adjusted for clustering in the data due to repeated measures on the same individuals and due to the intervention being delivered across multiple sites.

Content analyses will be carried out on the survey data provided by DBT therapists which will inform the implementation evaluation.

An economic evaluation will be performed to assess the cost-effectiveness of DBT versus treatment-as-usual for the treatment of adults with BPD who engage in self-harm. The cost-effectiveness analysis will compare the relative costs and outcomes (effects) of the DBT intervention. All relevant costs from the perspective of the health care provider will be identified, measured and valued. Direct health service use will be measured by means of a resource use questionnaire. The relevant health effects will be measured in natural health units using recognised scales, for example, the Beck Depression Inventory. In addition, Quality Adjusted Life Years (QALY) will be calculated with the EQ-5D-5 L utility scores. An incremental Cost Effectiveness Ratio (ICER) will be calculated comparing the relative costs and outcomes. The economic evaluation will be conducted in line with the eight-step framework put forward by Drummond et al. [57].

### Ethical principles

The participation in the study is voluntary. Participants are informed that if they decide not to participate in the study that this will not affect their treatment in any way. Participants are informed that they can withdraw their participation at any time without providing a reason. All participants are asked to sign an informed consent form.

### Ethics approval

Research ethics approval was sought and obtained from all relevant research ethics committees at the multiple sites of this research study.

## Discussion

The study protocol is outlined here to offer clinicians and researchers in publically funded health systems an opportunity to consider the methodological quality of this effectiveness study with a critical view. Publicly funded health systems may benefit from considering how the proposed protocol could be applied to outpatient mental health settings which treat individuals with emotional and behavioural dysregulation. There are a limited number of effectiveness studies looking at standard stage 1 DBT in public mental health settings. Research on effectiveness of DBT when implemented in a coordinated manner could provide an important contribution to improving routine mental health care for patients with BPD.

The protocol also offers clarity on quality, quantity and experience of participating in a coordinated implementation of DBT at a national level. This could potentially provide evidence on how best to overcome barriers to implementation in publicly funded health systems.

The economic evaluation which is carried out as part of this research study will provide important information on the cost of BPD illness to a public health service which will facilitate international comparison. In addition, evaluating the cost of implementing DBT in a coordinated manner in comparison to treatment-as-usual will provide support for the value of robust application of an evidence-based intervention which is also in line with best practice guidelines.

One of the limitations in previous research on DBT effectiveness is the variability in outcome measures utilised to assess clinical effectiveness. This protocol specifically matches outcome measures to treatment targets for DBT in an attempt to create a standardised battery of measures that can be applied across any research setting. Evidence on effectiveness, resource utilisation and feedback from both patients and therapists will shape systemic culture change in how we treat individuals with chronic self-harm and suicidal behaviours.

As this study is carried out in a publicly funded health system, this study considers how this treatment might be applied to multiple patient groups, in this case adults and adolescents. No study to our knowledge has reported on the effectiveness of the 16-week DBT programme as described by Miller and colleagues [28] for adolescents in community settings so it is anticipated that the results of this study will also contribute to this gap in the literature.

### Strengths and limitations

As this study is carried out in a real-world setting, DBT therapists do not have dedicated time to devote to research related tasks, as would be the case in a clinical research trial. Therapists are therefore participating in a voluntary capacity in addition to daily routine and regular professional practice. Given the nature of this comprehensive evaluation, it is anticipated that full compliance with the data collection protocol may not always be possible with competing clinical demands. In an effort to offset this risk, each team will be supported by a member of the coordinating team who will provide guidance and assistance with the research evaluation where possible. In addition, in real-world settings, therapists have a mixed qualification and skill set in relation to research related tasks in contrast to those specifically recruited to support clinical research trials.

One of the strengths of this study is that a dedicated research team has been recruited to coordinate and support this multi-site research evaluation. This was done in an effort to reduce experimenter bias which may be present if clinicians who are working with patients conduct data collection. It is anticipated that having a dedicated research team will also increase the reliability and quality of data collection at agreed time points, and will allow for follow-up data collection to maximise dataset completeness. Having a researcher assigned to each team who will support clinicians during the process of the study will also potentially increase response rates and dataset completeness. Feedback will be provided to each participating service and updates will be provided regarding recruitment status on a regular basis by the research team. At the end of the study, summary reports of the results for each individual site, in addition to the pooled data analysis, will be provided to each team to provide feedback to clinicians and aid service planning. The outlined difficulties with regard to real-world research needs to be managed if researchers and clinicians are to work towards increasing external validity i.e. the effectiveness of an intervention in a publicly funded health system implemented in a manner that is sustainable over time.

Although one of the challenges in conducting effectiveness studies is inclusion of data from a control group, the current study hopes to obtain a comparison group by utilising data collected from treatment-as-usual patients in areas with no DBT provision or where patients opted out of treatment. While real-world challenges prohibit a randomised controlled design in a community setting, there is still merit in having a comparison group who do not receive treatment for the purposes of the evaluation.

The comprehensive research evaluation includes both quantitative and qualitative feedback from multiple perspectives including that of the patient, their family member (in the case of adolescents) and their therapist. Such comprehensive and robust evaluation will serve to inform the real value of DBT as an intervention when delivered in a publicly funded health setting and will potentially serve to inform our system to refine and increase sustainability of service provision over time.

The advantage of having a multi-site study is that it yields a larger sample size. A strength of this study is that we have sufficient power in both the adult and adolescent populations to answer the research question as to whether DBT, if delivered in a coordinated manner, will produce positive outcomes for patients in a publicly funded health system.

This study will potentially provide evidence to endorse DBT as an evidence-based treatment that can be effectively delivered in community settings for high risk individuals. This approach will potentially lend itself to better coordinated interventions and public health system changes that may reduce the need for repeated emergency department attendances and protracted periods of acute hospitalisations.

## Abbreviations

BDI: Beck Depression Inventory
BHS: Beck Hopelessness Scale
BPD: Borderline Personality Disorder BSL Borderline Symptom List
CBT: Cognitive Behaviour Therapy
DBT: Dialectical Behaviour Therapy
NICE: National Institute for Health and Care Excellence
QOL: Quality of Life

## Footnotes

1 In 2013, recommendations by BiDBT allowed up to a maximum of ten members per DBT team. This has since been revised and current training requirements allow a maximum of eight team members.
