# Correction to: Dialectical behaviour therapy for treating adults and adolescents with emotional and behavioural dysregulation: study protocol of a coordinated implementation in a publicly funded health service

**DOI:** 10.1186/s12888-018-1694-y

**Published:** 2018-05-07

**Authors:** Daniel Flynn, Mary Kells, Mary Joyce, Catalina Suarez, Conall Gillespie

**Affiliations:** 1 Cork Mental Health Service, Cork Kerry Community Healthcare, Health Service Executive, St Finbarr’s Hospital, Cork, Ireland; 2 National Suicide Research Foundation, Western Gateway Building, University College Cork, Cork, Ireland

## Correction

Upon publication of the original article [1] it was highlighted by the authors that there was just one error in the manuscript in the ‘Sample size’ subsection of the Methods/Design. The current text reads:

“It is anticipated that there will be a total of 442 participants across 16 sites in this research study over a four year period. Of the 312, it is estimated that 120 will be adults with a primary diagnosis of BPD attending Adult Mental Health Services across eight study sites.......”

The correct text should have “312” replaced with “442” so that it reads:

“It is anticipated that there will be a total of 442 participants across 16 sites in this research study over a four year period. Of the 442, it is estimated that 120 will be adults with a primary diagnosis of BPD attending Adult Mental Health Services across eight study sites.......” This has since been formally noted in this Correction article.

## References

[1] Flynn D, Kells M, Joyce M, Suarez C, Gillespie C (2018). Dialectical behaviour therapy for treating adults and adolescents with emotional and behavioural dysregulation: study protocol of a coordinated implementation in a publicly funded health service. BMC psychiatry.

